# Practical Points in the Medical Care, and Nursing of Children

**Published:** 1873-05

**Authors:** Jerome Walker

**Affiliations:** Physician to the Sheltering Arms Nursery, Brooklyn


*Practical Points in the Medical Care, and Nursing of Children.* I. *Natural Feeding.* By JEROME WALKER, M.D., Physician to the Sheltering Arms Nursery, Brooklyn.

Although experimental researches and practical conclusions are demanded in these times, and the substitution of positiveness for uncertainty is carefully being attained in the different branches of medicine, yet the absolutely *necessary* points in the medical care of children are only *tacitly* acknowledged by the majority of medical men to be important, for the carrying out of these points requires "too much care and time." Medical men may admit the force and truth of certain conclusions, but fail to make them of service because great patience, much study, untiring energy, and absolute love of children, are essential to their proper care.

In an effort to systemize and to obtain rules for my own guidance, certain deductions forced themselves upon me, and I was surprised and pleased to find most of them arrived at, and some enlarged upon by Eustace Smith, of London, in his admirable work on the "Wasting Diseases of Infancy and Childhood." As this work is the only one which enters into anything like a full discussion of the "points," I mention the fact, that I may not be suspected of plagiarism.

Some time since, the following questions were sent to Drs. Jacobi, J. Lewis Smith, E. S. Dunster, and J. B. Reynolds, men who from large experience, opportunities for observation, and particular attention to the care and treatment of children, were believed to be reliable exponents of all ascertained facts—viz.:

1. If mother and child *seem healthy* and child is being *nursed*, at what time do you advise the use of other food than mother's milk?
2. If other food be used, *what* is it, *how prepared* and *what* are its *ingredients?*
3. When a nursing woman becomes pregnant, do you advise artificial feeding?
4. State results of trials with so-called substitutes for mother's milk.
5. Is *dentition* the signal for weaning, or the employment of solid food?
6. Do you insist upon stated periods for nursing or feeding, as the case may be, during infancy?
7. In case of *weakly* children, or when it is thought best that the mother should not nurse, do you advise wet-nursing?
8. Is the milk of wet-nurse to be of the same age as child has been accustomed to?
9. State experience with milk older or younger.
10. State preference for one animal's milk over another.
11. Opinion of condensed milk.
12. In dilution of milk, how much water do you use?

The substance of this paper (with others which will follow in due course), shall be the consideration of the above questions. For the purpose of impressing more forcibly the "practical points," on the minds of my readers, these "points" will be discussed under the following headings:

1. *Natural* feeding of children.
2. *Artificial* feeding of children.

Natural Feeding.—Physicians, as a rule, believe with Dr. Jacobi "that in *theory* everybody agrees if it were only on philosophical or religious principles, that babies ought to be reared on breast-milk." The inconsistent *practice*, so common in all stations of life, may be traced oftentimes to the careless teaching, or the absence of teaching, of medical advisers. With the public and the profession the extra care and attention demanded by the weakness and tenderness of infancy, give rise, first to carelessness, next uncertainty, and finally recklessness.

Much has been written upon the importance of natural feeding. Such works as those of Eustace Smith, West, Vogel, Bouchut, Meigs, and Pepper, and lectures thorough and systematic as those of Dr. Jacobi, *must* help to clear away the indifference so prevalent. "Every woman," says Stoll, "who can bear a child can suckle it, at least during the time she keeps her bed." "This short time," writes Eustace Smith, "is an advantage to the infant not to be despised. All mothers ought to try to nurse, partly for their own sakes, and partly for the child's." "How short soever the period may be during which the mother is able to suckle her child, it is very desirable that she should nurse it during that period, and also that her milk should then constitute its only food; for this secretion is especially adapted to the feeble powers of the digestive organs soon after birth, and it is far from easy to find a substitute," says West. Cazeau gives certain vague objections to mothers nursing, viz.—"Any formal contra-indication, such as a very lymphatic constitution, presence of skin disease, or of predisposition, hereditary or otherwise, to phthisis," and the ease with which formal contra-indications are thereupon seen,gives an impetus to unnatural feeding.

The fact is, most of us are too apt to allow our sympathy, from fears of supposed injury to mother or child from nursing, to take the place of calm, deliberate judgment, forgetting that experience has taught, that with children who may from necessity be compelled to live on artificial food, those generally survive and grow who have had a chance at breast-milk, for even a short time only, following birth. I believe this to be a generally accepted fact, especially *noticed* in institutions for the young.

The *prohibition* of nursing from birth, unfortunately must be left to the judgment of each individual practitioner. The causes for a *cessation* of nursing are plainly—its bad effect on the mother, the poorness and insufficiency of her milk, and the unsatisfactory condition of the child, as shown by its general appearance, mode of suckling, and gradual decrease in weight. The first is apparent to every one, the second can only be determined by careful comparison between healthy and sickly children, and the third ascertained by any decided departure from results of experiments obtained by Chaussier and Quetelet, viz.: average increase in weight for first 9 days after birth, $\frac{1}{4}$ lb., first 14 days, $\frac{1}{2}$ lb., and probably not reaching 1 lb. till 28th day; and of those made by M. Guillot at the Foundling Hospital, Paris, before and after nursing, when he finds that $2\frac{1}{4}$ lbs. of milk, avoirdupois, is the smallest quantity that suffices for the nourishment of a healthy infant during the first month of its existence, or $2\frac{1}{2}$ ounces for each act of suckling.

E. Von Siebold found "that the weight, for a few days after birth, diminished."

Though these experiments are necessarily limited, yet the rough approximation has its value.

Examination of Milk.—An examination of the milk is necessary, either in the selection of a wet-nurse, or in deciding upon the cessation of nursing for the mother, but the health of the two respective mothers and their children forms the truest guide. Four points are to be considered in this examination, and as to the effect of the milk upon the child :

1. Its suitableness and sufficient quantity.
2. Its suitableness and insufficiency.
3. Its poorness and abundance.
4. Its poorness and scarcity.

Each condition gives rise to certain phenomena in the child.

With the quality and quantity of the milk—as they should be—the child is a healthy one, i. e., with limbs rounded and well filled out, the skin clear, fresh looking, and of a light rosy tinge. Such a child sleeps a good part of the time, and when awake, is bright, nurses easily and quietly, and when through nursing leaves the breast. During twenty-four hours the bowels move two or three times, and the motions are copious and of a bright yellow color.

"When the food is suitable but insufficient, the child loses the plumpness, the muscles become flaccid and soft. His face is pale and thin, bones become prominent. He is peevish, takes the breast, at first ravenously, and then at intervals stops to cry piteously. The skin is moist and cool. The fontanelle level or slightly depressed. At night he is very fretful. In the day time may lie quietly but sucks his fingers." There is an irregularity in the fecal movements, as to the time and character of the motions.

"With the food poor but abundant, the child is usually very quiet and drowsy, not uncommonly sleeps at the breast." Eustace Smith believes this last to be a sure sign that the milk is thin and serous. "The bowels are usually costive, and the motions rather solid although otherwise natural."

When the fourth condition is present, "we find the symptoms of defective nutrition combined with the symptoms of indigestion. The child begins to waste, grows dull. The skin, at first moist, then becomes dry, except about the head. Fontanelle is depressed. The face and body, at first pale, assume a dirty yellow hue, which may or may not disappear. The bowels irregular at first, constipation alternating with diarrhœa, then diarrhœa is constant. Flatulence is common. Thrush, inflammation and ulcerations of the skin often appear. Ulceration of the corneæ may occur.

Now that the condition of the child has been considered, let us turn our attention to the milk more especially.

What is healthy milk and what unhealthy ?

HEALTHY MILK.—The composition of healthy milk, according to Bouchut, is:

| | At 1st Month. | At 24th Month. |
|---|---|---|
| Sp. gravity, | 1033.11 | 1030.81 |
| Water, | 872.99 | 876.55 |
| Solid constituents, | 127.61 | 123.45 |
| Sugar, | 43.13 | 41.33 |
| Butter, | 34.05 | 41.47 |
| Casein, | 48.26 | 37.32 |
| Salts, | 1.57 | 1.33 |

Lehman says, "women's milk in general is of more bluish white color than cows or any animals, is sweeter, has a strong alkaline reaction."

Simon found by analysis made every ten days for six months, that at the commencement of nursing, casein is at its minimum, then rises to nearly a fixed proportion, the salts increasing with it. Second, sugar at its commencement is at its maximum, then diminishes. Third, butter is variable. These analyses agree with those of Vernois, Becquerel and others. Pelegot and Reiset found more fat generally toward the close of nursing. These practical conclusions form the guide in the preparation of any food for infants.

*Tests.*—To prove the properties of milk, certain tests are useful. Those of Vogel are explicit and concise, viz.:

1. Fill graduated galactometer with milk, let it stand covered for twenty-four hours, then the cream should at least comprise three lines of the glass in thickness.
2. Test with blue and yellow paper.
3. Taste, which should be insipid and slightly sweet.
4. The microscope.

"If nurse has been confined more than eight days, colostrum corpuscles and epithelial cells should not be present at all, or in small quantities, milk globules of unequal size or too many." "In pure milk," says M. Doune, "nothing but milk globules are seen." The same writer also shows human milk to be alkaline. I have not been able to find any reported case of acidity in human milk.

UNHEALTHY MILK.—Unhealthy milk may be inferred from its scarcity of cream, change in color and taste, generally slight, but principally from microscopic examinations.

In 1853 Vogel* first discovered vibriones in human milk. There was no fermentation, as the milk was alkaline. The child had all the appearances produced by malnutrition. From 1854 to 1861 Dr. Gibb,† in an examination of many hundred

* *Lancet*, 1854.

† Archiv. of Med., No. 7, on the saccharine fermentation of milk in the human breast.

specimens of human milk, found two genera of animalculæ where the general health was disordered during lactation, or where lactation was prolonged, or an insufficient quantity of milk secreted, found in all periods of lactation, milk varying in color, sp. gr. 1024 to 1035, and almost invariably neutral or alkaline. The children, as a rule, were badly nourished. The Doctor believes that the vibriones themselves are not hurtful, but produce a bad change in the milk. Inflammatory globules have been found by Lehman and others, when there was any acute affection in the mother. Vernois and Becquerel * found "an excess of butter or caseine always to accompany an ill state of the nursling."

We are then to decide upon the continuance or cessation of nursing by the mother, and in the selection of a wet-nurse as well, first by tests, as before described; second, the history and present constitution of the mother; third, the condition of the child.

No writer, as far as I can ascertain, indorses the assertion of M. Doune, "that there is an almost constant relation between the composition of the fluid during pregnancy and that of the milk afterwards." Cazeau thinks "that the results obtained by M. Doune may enable us to form a *probable* diagnosis as to what each woman's milk *is to be* in quantity and quality."

If the milk after examination is found to be simply poor and small in quantity, then generous diet, suitable tonics, attention to hygiene, use of warm drinks, especially at night, a moderate use of malt liquor, applications of electricity, poultices of castor oil plant leaves to the breasts, and the use of the fluid extract of the same internally, as recommended by Dr. Wm. Gilfillan, of this city, are often efficacious. To these means may be added, as a drink through the day, or better still, on going to bed, and through the night, if necessary, a very pleasant mixture of tr. ginger (or Brown's ess. of Jam. ginger), and sweetened milk; about one teaspoonful of ginger to tumbler of milk, and sweetened to taste. The use of the air-pump, as designed by the patentees of the so-called air-treatment, is probably useful. Cazeau found one nurse whose milk was sensibly increased several times by eating ground lentils. Vernois, Becquerel, and others, found that a vegetable diet made the milk richer in butter and sugar, while the solid constituents were increased by mixed food.

The suggestions of Eustace Smith are valuable. "If the mother has been over-stimulated by a too rich diet, diminishing the quantity of her food, and the administration of a gentle saline purgative, generally produces a plentiful supply of milk. Feverishness, from whatever cause, will necessarily tend to diminish the lacteal as well as the other secretions of the body.

* April, 1853. *Brit. and For. Med. Review.*

In such cases, therefore, increasing the quantity of food would have an effect the very opposite of that which it is intended to produce. *All* of the means in our power should be faithfully tried before we take upon ourselves the *great responsibility* of changing the food of infants.—*The Sanitarian.*

